# Impaired recognition of facial expressions of emotions in refugees: The role of war‐related trauma

**DOI:** 10.1002/jts.70015

**Published:** 2025-09-14

**Authors:** Edita Fino, Denis Mema, Maria Ida Gobbini

**Affiliations:** ^1^ Department of Psychology University of Bologna Bologna Italy; ^2^ Brückenkomponente Albanien Deutsche Gesellschaft für Internationale Zusammenarbeit GmbH Tirana Albania; ^3^ Department of Medical and Surgical Sciences, St. Orsola‐Malpighi Hospital Alma Mater University of Bologna Bologna Italy; ^4^ IRCSS Institute of Neurological Sciences Bologna Italy

## Abstract

Exposure to traumatic events is associated with biases in the perception of emotional facial expressions. By bridging research on trauma exposure and emotion recognition, the present study investigated the impact of war‐related trauma on the recognition of facial expressions of emotions in a sample of war trauma–exposed refugees (*N* = 108) from West Asian countries. Through a forced‐choice facial emotion recognition experiment, we assessed how trauma exposure and face gender influenced accuracy and biases in identifying six primary emotions. Participants judged facial expressions of anger, sadness, fear, disgust, surprise, and happiness displayed by a set of 240 faces corresponding to 20 female and 20 male models from the Karolinska Directed Emotional Faces dataset. Expressions consisted of short videos showing each face's transition from neutral to full emotion. The results showed impaired recognition of negative emotions, with fear being the least accurately recognized emotion, suggesting the avoidance of negative affective states as a coping mechanism putatively associated with war‐related trauma. For main effects, partial eta‐squared effect sizes ranged from .159 to .573, and effect sizes for interaction effects ranged from .027 to .189, with most effects being in the medium‐to‐large range. Furthermore, the biases in emotion recognition observed in the present study may reflect gender stereotypes and social norms that shape how individuals perceive and interpret emotional expression in men and women.

The ability to accurately identify and interpret facial expressions of emotions is fundamental for effective communication and interaction, playing a crucial role in individuals’ adaptation to their social environment (Ekman, 2003). Emotion recognition serves as the foundation for more complex social skills and is associated with positive social interactions, whereas difficulties in perceiving and understanding others’ emotional states can impair social functioning, increase negative affect, and contribute to psychopathology (for a review, see Trentacosta & Fine, 2010). Research has linked exposure to traumatic events with biases in the perception of emotional facial expressions, with most studies indicating impairment in emotion recognition (Castro‐Vale et al., 2020; Motsan et al., 2022), although some reports suggest a facilitation effect for specific emotions (e.g., anger in ambiguous faces; Gebhardt et al., 2017). Although the type of traumatic event an individual experiences may shape patterns of emotion recognition, relatively few studies have explored the effects of war‐related trauma on refugees, instead focusing primarily on veterans or individuals with combat experience (Poljac et al., 2011; Umiltà et al., 2013). The impact of war‐related trauma on displaced civilians’ ability to accurately interpret emotional expressions remains understudied despite rising numbers of individuals displaced due to war and civil conflict (Fino, 2024). Trauma can alter how individuals interpret emotional and social cues, which can hinder their ability to successfully navigate social relationships and adapt to unfamiliar cultural environments (Hayes et al., 2012). Understanding how war‐related trauma affects the perception of emotions among displaced civilians may be crucial to informing psychosocial interventions aimed at facilitating their social adjustment and integration into new societies.

In the context of trauma, most studies of emotion recognition have focused on the prevalence of psychopathology and trauma‐related disorders, including posttraumatic stress symptoms (PTSS), suggesting that there may be an association with impairments in cognitive, social, and emotional regulatory systems (Fino, Mema, & Russo, 2020; Shonkoff, 2016). Trauma exposure and PTSS have been generally related to emotion recognition impairment (Schönenberg & Abdelrahman, 2013; see also Gebhardt et al., 2017). For instance, Poljac et al. (2011) found a reduced recognition accuracy of faces displaying fear or sadness in participants with PTSS compared with healthy controls. Other studies have also demonstrated an impaired recognition of sadness among combat‐ and violence‐exposed youth compared with civilians and controls (Ardizzi et al., 2013; Umiltà et al., 2013). Impairment in emotion recognition following traumatic events seems to extend beyond negative emotions. Recent findings (Castro‐Vale et al., 2020; Rutter et al., 2022) suggest lower accuracy for both positive and negative emotions during an emotion recognition task among individuals with a history of cumulative trauma exposure and PTSS compared to nontraumatized healthy controls.

A failure to recognize emotional expressions in individuals exposed to trauma has been linked to reduced autonomic reactivity and lower respiratory sinus arrhythmia, indicating a weakened stress response and less variation in heart rate when observing others’ facial expressions (Ardizzi et al., 2013). Similarly, other studies report a weakened neural processing (i.e., reduced late positive potentials; LPPs) of social signals of threat (Macatee et al., 2020; MacNamara et al., 2013). Additionally, higher levels of alexithymia, characterized by difficulty describing, identifying, and differentiating one's feelings, as well as a tendency to focus attention externally rather than internally, have been associated with reduced attention toward faces displaying negative emotion in the context of posttraumatic stress (Passardi et al., 2019). Reduced processing of threatening stimuli in trauma‐exposed individuals may reflect an adaptive response in the short term, as it may prevent the reactivation of traumatic memories and inhibit emotional arousal. Avoiding emotional states related to trauma may indicate a disengagement from affective cues, both internal and external, which may be associated with difficulty identifying and describing emotions. Recognizing emotions in other individuals partly involves simulating those states via mirror neuron systems; thus, the chronic suppression or avoidance of emotional experiences may lead to lower neural responsiveness to this type of simulation, resulting in reduced emotional resonance with other individuals’ states (Ardizzi et al., 2013). Research shows that people who are exposed to cumulative traumatic events are more likely to have blunted cardiovascular reactions to trauma reminders (Lee et al., 2022) and to experience numbing or avoidance instead of a more typical hyperarousal stress response. This is in line with research showing that the PTSS avoidance cluster seems to be a strong predictor of impairment in emotion recognition (Mazza et al., 2012).

Although much of the literature on emotion recognition following trauma has focused on a few negative emotions (for a review, see Seidemann et al., 2021), disgust has been less investigated despite its relevance in the war context, which may involve exposure to injury, death, mutilation, unclean conditions, or moral violations that may trigger strong disgust responses (Rozin et al., 2008). Research suggests that disgust may also play a role in the development and maintenance of posttraumatic stress disorder (PTSD; Badour & Feldner, 2018). Moreover, most studies have focused mainly on war veterans (Poljac et al., 2011) and individuals with combat experience (Umiltà et al., 2013), with civilians exposed to war‐related trauma often treated as a control group, though individuals with combat experience represent a small percentage of the war‐exposed population. The way exposure to war‐related trauma influences civilians’ ability to recognize emotions in the facial expressions of others has been less examined. In the present study, we addressed this gap by bridging research on war‐related trauma exposure and facial emotion recognition and evaluated the recognition of six basic emotions (fear, anger, sadness, disgust, surprise, and happiness) in a sample of war trauma–exposed refugees and asylum seekers from West Asian countries. We further examined the effect of face gender on emotion recognition patterns.

## METHOD

### Participants and procedure

Participants (*N* = 108) were refugees and asylum seekers (*n* = 96 men, age range: 31–65 years) from West Asian countries who were residing in the National Reception Center for Refugees (NRC) in Tirana, Albania, during 2018–2019. Individuals were recruited with the assistance of the local Association for Refugee and Migrant Services (RMSA) to complete a questionnaire reporting on sociodemographic information and experiences of premigration trauma exposure, as well as an emotion recognition task. Participants were told that participation was voluntary, and they could withdraw from the study at any time with no consequences, and all participants gave verbal consent. The protocol complied with the Declaration of Helsinki II, and ethical approval was obtained by the joint Review Committee of the RMSA and the NRC (21.01.2017).

### Measures

#### War‐related trauma exposure

War‐related trauma was assessed using the Harvard Trauma Questionnaire–Part I (HTQ‐I; Mollica et al., 1992), which includes a checklist of 43 potentially traumatic war‐related events (e.g., physical violence, environmental hardships). Participants indicated whether they had experienced or witnessed each event (e.g., “forced to flee your country,” “witnessed someone being physically harmed”) using a “yes” or “no” response format. Each endorsed item (experienced or witnessed) was scored as 1, and a cumulative total score was calculated to reflect overall trauma exposure. The HTQ is one of the most robust and widely validated tools for refugee trauma assessment (for a review, see Sigvardsdotter et al., 2016), with good psychometric support from studies across multiple languages and cultural settings (Kleijn et al., 2001; Lhewa et al., 2007; Rasmussen et al., 2015; Shoeb et al., 2007).

#### Emotion recognition task

Stimuli were taken from the Karolinska Directed Emotional Faces database (KDEF‐dyn; Calvo et al., 2018). The KDEF‐dyn is a validated dynamic version of the original KDEF database 9Lundqvist et al., 1998) in static format that contains 240 video‐clip versions (1,033 ms duration) of the original KDEF face stimuli, corresponding to 40 models (20 women and 20 men) with six emotional facial expressions (happiness, anger, sadness, surprise, fear, and disgust). The videos show the transition from neutral to full emotional expression (see Calvo et al., 2018) and were displayed on a computer screen using OpenSesame software. A total of 240 video clips were presented (40 models for six expressions) in six blocks of 40 trials each, with a short break after each block. Block order was counterbalanced, and trial order and type of expression were randomized within each block. The trial sequence consisted of an initial 500‐ms central fixation cross, followed by a video clip showing an emotional facial expression for 1,033 ms. After face offset, six small boxes arranged horizontally appeared at the bottom of the screen, showing labels for the six emotions from which participants had to identify the correct facial expression by right‐clicking with the mouse on the corresponding box. There was a 1,500‐ms intertrial interval.

### Data analysis

In a within‐subjects experimental design, we measured recognition accuracy (i.e., responding “happy” when the face stimulus was intended to convey happiness) and accuracy bias (i.e., selecting any other emotion expression when the face shows “happy”) for each emotion expression. The responses for each facial expression were transformed into proportions. Multiple repeated‐measures analyses of variance (ANOVAs) were performed on scores of emotion recognition accuracy and emotion recognition bias, with emotional facial expressions (happiness, sadness, anger, fear, disgust, and surprise) and face gender (men vs. women) as within‐subject factors. Significant interactions were further examined using Bonferroni‐corrected post hoc tests. Multiple regressions were performed to evaluate the associations between trauma exposure and emotion recognition accuracy. There were no missing data, and all analyses were performed using SPSS (Version 24).

## RESULTS

### Emotion recognition accuracy

**FIGURE 1:**
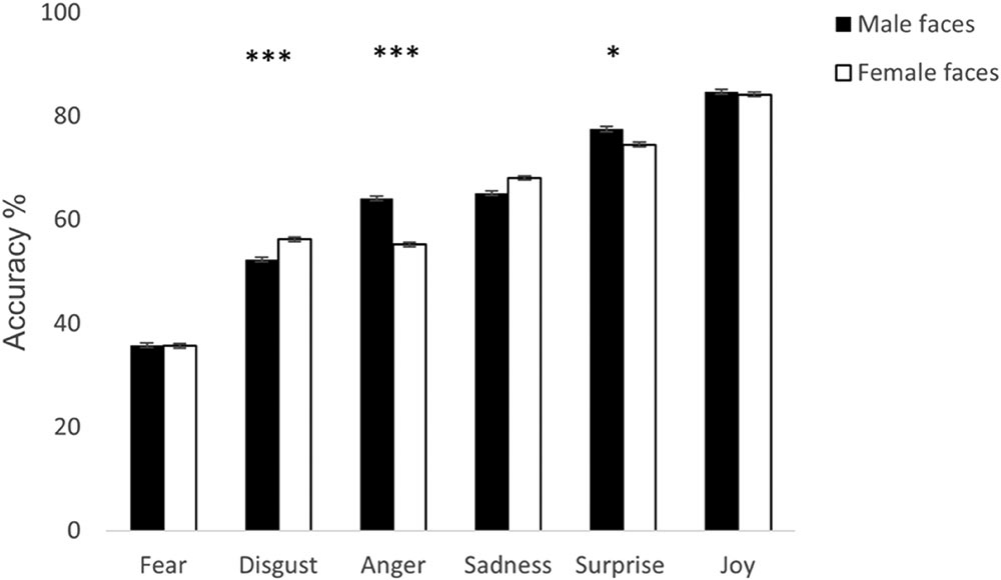
Emotion recognition accuracy for male and female faces *p < .05. **p < .01. ***p < .001.

A summary of the main and interaction effects for the ANOVAs is provided in Table 1. The percentage of participants who correctly identified each expression as the one intended by the model is shown in Figure 1. Happy expressions (*M* = 86.1%) were identified more accurately than all other expressions, followed by expressions of surprise (*M* = 77%). The least accurately recognized expression was fear (*M* = 37.7%), followed by disgust (*M* = 55.5%), anger (*M* = 63.7%), and sadness (*M* = 68.5%). Angry expressions were identified more accurately when posed by male faces (*M* = 64.1%) than female faces (*M* = 55.2%), *p* < .001. Disgusted expressions were identified more accurately when posed by female faces (*M* = 56.2%) than male faces (*M* = 52.3%), *p* < .001, and the same was true for sad expression (female: *M* = 70.0%, male: *M* = 67.0%), *p* = .048. Recognition accuracy rates were significantly different for all emotions, *p*s < .001. Anger and sadness were similarly recognized when posed by male faces, *p* = .642, whereas anger and disgust were equally recognized when posed by female faces, *p* = .391. Multiple regressions were conducted with trauma exposure as the predictor variable and emotion recognition accuracy rates for each emotional expression as dependent variables, with the results showing that higher levels of trauma exposure were associated with decreased accuracy in recognizing both negative and positive emotion expressions, as indicated by the negative standardized beta coefficients, *p*s < .05. The results of the multiple regression analysis are summarized in Table 2.

**TABLE 1:** Results of multiple analyses of variance facial emotion recognition accuracy and error rates, with emotion expression and face gender as within‐subjects factors

Effect type	Factor(s)	Statistical test	*p*	η_p_^2^
*Dependent variable: Emotion recognition accuracy*				
Main effect	Emotion expression	*F*(5, 102) = 127.599	< .001	.544
Interaction effect	Emotion Expression x Face Gender	*F*(5, 102) = 12.940	< .001	.108
*Dependent variable: Emotion recognition bias*				
Angry faces				
Main effect	Emotion expression	*F*(4, 103) = 115.338	< .001	.519
Main effect	Face gender	*F*(1, 106) = 51.183	< .001	.324
Interaction effect	Emotion Expression x Face Gender	*F*(4, 103) = 5.298	< .001	.047
Disgusted faces				
Main effect	Emotion expression	*F*(4, 103) = 143.390	< .001	.573
Main effect	Face gender	*F*(1, 106) = 9.413	.003	.081
Interaction effect	Emotion Expression x Face Gender	*F*(4, 103) = 10.092	.008	.086
Fearful faces				
Main effect	Emotion expression	*F*(4, 103) = 95.962	< .001	.473
Interaction effect	Emotion Expression x Face Gender	*F*(4, 103) = 24.901	.037	.189
Sad faces				
Main effect	Emotion expression	*F*(4, 103) = 20.189	.001	.159
Interaction effect	Emotion Expression x Face Gender	*F*(4, 103) = 12.367	< .001	.104
Surprised faces				
Main effect	Emotion expression	*F*(4, 103) = 34.688	< .001	.245
Interaction effect	Emotion Expression x Face Gender	*F*(4, 103) = 12.159	.001	.102
Joyful faces				
Main effect	Emotion expression	*F*(4, 103) = 29.081	.001	.214
Interaction effect	Emotion Expression x Face Gender	*F*(4, 103) = 3.010	.018	.027

**TABLE 2:** Results of multiple regressions, with trauma exposure as the predictor variable, and recognition accuracy rates for each facial expression and stratified by face gender

Variable	β	*SE*	95% CI	*p*
Fearful faces	−.642	.042	[−.495, −.310]	< .001
Male faces	−.552	.054	[−.474, −.261]	< .001
Female faces	−.642	.047	[−.495, −.310]	< .001
Angry faces	−.498	.106	[−.841, −.419]	< .001
Male faces	−.477	.056	[−.422, −.201]	< .001
Female faces	−.482	.056	[−.430, −.207]	< .001
Disgusted faces	−.361	.104	[−.620, −.207]	< .001
Male faces	−.278	.052	[−.257, −.052]	.004
Female faces	−.626	.093	[−.955, −.585]	< .001
Sad faces	−.265	.102	[−.489, −.086]	< .001
Male faces	−.285	.059	[−.296, −.063]	.003
Female faces	−.201	.051	[−.208, −.007]	.037
Surprised faces	−.324	.11	[−.603, −.169]	.001
Male faces	−.277	.063	[−.311, −.062]	.004
Female faces	−.352	.052	[−.302, −.097]	< .001
Joyful faces	−.331	.112	[−.623, −.180]	< .001
Male faces	−.304	.06	[−.315, −.078]	.001
Female faces	−.333	.056	[−.317, −.093]	< .001

*Note*: CI = confidence interval.

### Emotion recognition bias

Table 1 provides a summary of the main and interaction effects for all ANOVAs. Facial expressions of anger were most often misclassified as disgust (*M* = 23.9%) or sad expressions (*M* = 10.3%). Angry faces were more often misclassified as fearful and sad for female compared to male faces, *p*s  = .001–.006. Faces conveying disgust were most often misclassified as angry (*M* = 23.9%) or sad (*M* = 10.28%). Disgust was more likely to be misclassified as anger in male compared to female faces, whereas it was more often misclassified as joy in female faces, *p*s = .001‐ .002. Expressions of fear were most often misclassified as surprised (*M* = 26.8%) or sad (*M* = 14.2%). Fearful female faces were more likely to be misclassified as surprised or joyful, *p*s < .001), whereas fearful male faces were misclassified as sad and disgusted, *p*s = .001‐ .020 Sad expressions were misclassified most often as fearful (*M* = 9.1%) or disgusted expressions (*M* = 8.5%), and they were more likely to be misclassified as disgusted, happy, or surprised when conveyed by male faces and as angry when conveyed by female faces, *p*s = .001– .021. Surprised faces were misclassified most often as fearful (*M* = 11.9%) or happy (*M* = 4.5%). Male surprised faces were more likely to be misclassified as fearful, whereas female surprised faces were more likely to be misclassified as angry and joyful, *p*s  = .001–.030. Lastly, happy faces were most often misclassified as fearful and sad in male compared to female faces, *p*s  = .001–.031. See the Supplementary Material for more details on facial expression misclassifications for each emotion and male and female faces respectively (see Supplementary Figures S1–S6).

## DISCUSSION

Previous research has linked blunted autonomic, cardiovascular (Ardizzi et al., 2013; Lee et al., 2022), and neural responses (MacNamara et al., 2013) to emotional facial expressions with impaired emotion recognition in trauma‐exposed individuals, suggesting it may reflect coping mechanisms of avoidance or denial of emotional states putatively associated with war‐trauma events, which may explain the difficulty of identifying the same emotions when witnessed in others (Mazza et al., 2012). Our results are in line with such evidence, considering that the negative emotions of fear, disgust, anger, and sadness were the least accurately recognized among participants in the present study. According to Haidt and Keltner's (1999) 70%–90% criterion, which is used as a benchmark for validating facial expressions in emotion in recognition studies, our participants correctly recognized happy (*M* = 86.0%) and surprised expressions (*M* = 77.0%) compared sad (*M* = 68.5 %), angry (*M* = 63.7%), disgusted (*M* = 55.5%), and fearful expressions (*M* = 37.7%). Although this pattern of recognition accuracy converges with previous findings on static expressions among the general population (Nelson & Russell, 2013), the accuracy rates in our sample were lower for negative emotions when compared to studies using the same dynamic expressions (Calvo et al., 2016, 2018). We found especially low accuracy rates for fear, which may be due to fear being a prevalent emotional reaction in war‐exposed settings. However, this pattern of results may also reflect cross‐cultural variations in emotional expression and recognition, potentially shaped by broader sociocultural norms and practices. In support of this view, Nelson and Russell (2013) found that although happiness is consistently recognized across cultural and linguistic boundaries, negative emotions tend to be identified less accurately by non‐Western observers than Western observers. Furthermore, our findings suggest a dose–response effect such that higher degrees of trauma exposure exacerbate impairments in recognizing both negative and positive emotions. This is partially in line with studies on war‐exposed veterans and soldiers (Ardizzi et al., 2013; Poljac et al., 2011; Umiltà et al., 2013) that have reported lower accuracy in negative emotion recognition in these individuals compared to civilians or controls. Our study extends previous evidence by examining a wider range of emotions (i.e., happiness, surprise, and disgust) in a war trauma–exposed civilian population and by further exploring the effects of the gender of the face conveying the emotions. The findings suggest that the face gender modulates accuracy in emotion recognition in war trauma–exposed individuals such that angry expressions were more accurately identified in male compared to female faces, whereas disgust and sadness were most accurately reported for female compared to male faces. This is in line with gender differences reported in previous studies with the general population (Calvo & Lundqvist, 2008), suggesting differences in social norms that tend to reinforce different emotional expressions in men and women (Plant et al., 2000). For instance, overt expressions of anger are more associated with masculinity and dominance, and, thus, anger is a more readily identifiable emotion in men (Hess et al., 2000). The higher accuracy of angry male facial expressions may also reflect the fact that men are usually the main aggressors in war‐torn areas, and a higher sensitivity to threat detection would be functional for survival. Indeed, previous research (Gebhardt et al., 2017) has shown a facilitation effect of male ambiguous angry expressions among soldiers with PTSD following deployment. Women, on the other hand, are often expected to suppress anger and display emotions like fear, disgust, and sadness more openly. The observations in our study of the overmisattribution of disgust as anger in male compared to female faces and overmisclassification of fear and sadness to the detriment of anger in female compared to male faces further extend previous findings from the general population to a war trauma–exposed civilian group.

The analysis of errors provides further insight into the association between what participants saw and what they reported when making mistakes. The systematic patterns in misattributions of emotional expressions, particularly between fear and surprise (and vice versa) and between anger and disgust (and vice versa), with sadness being misperceived as fear or disgust, is in line with previous findings on emotion recognition bias in general populations (Calvo & Lundqvist, 2008; Recio et al., 2013). However, our results add another layer by examining the modulating effect of face gender. Specifically, when incorrectly labeling fear, participants were more prone to indicate that they saw surprise or happiness in female compared to male faces, whereas participants indicated the perceived sadness and disgust in male compared to female faces. On the other hand, surprise expressions were mostly misattributed to fear for male compared to female faces, whereas surprise was misclassified as joy or anger in female compared to male faces. These results may reflect social stereotypes associated with emotional expression in men and women. Misattributing fear to joy and surprise in female faces may reflect a positivity bias in interpreting women's expressions, aligning with the stereotype that women should be warm, pleasant, and socially engaging (Hess et al., 2005). On the other hand, the tendency to mislabel fear as sadness in men may be linked with the stereotype that men should not display vulnerability (i.e., fear), hence sadness may be perceived as a more acceptable “weak” emotion for men than fear, as it might be associated with stoicism, loss, or internal struggle (Barrett & Bliss‐Moreau, 2009). The surprise–fear misattribution that was more likely in male faces may also be understood in light of the war context, where misinterpreting men's surprise as fear might indicate an overestimation of men's distress or the assumption they are reacting to danger. Hence, when people see a surprised expression on a male face, they might default to fear because it may be linked to an external threat or danger, which fits better with masculine roles of being alert and protective of others. On the other hand, misinterpreting fear as surprise and joy in female faces in the war context resonates with research showing that women are more prone to respond to threat by social affiliation and positive social contacts, also known as the *tend‐and‐befriend* response (Fino, Fino et al., 2020; Taylor, 2012), which allows for experiencing positive emotions during adversity.

It should be noted that overall, when participants made incorrect identifications of fear, anger, and disgust, the second most likely misattributed emotion was sadness. A bias toward sadness misattribution has been previously reported in war trauma–exposed individuals (Umiltà et al., 2013), but the effect of face gender, as far as we know, has not been examined before. Our findings suggest that when participants misinterpreted sadness, they tended to see anger in women and disgust or surprise in men. Although women are stereotypically associated with emotions related to vulnerability, such as sadness and fear, they are also discouraged from openly expressing anger (Hess et al., 2000). Hence, people may default to anger when they see sad female faces, reinforcing the stereotype of women as “overreacting” or expressing “passive‐aggressive anger” (Barrett & Bliss‐Moreau, 2009). Men, on the other hand, are socially discouraged from openly expressing sadness, so observers may misattribute this emotion as disgust, which aligns better with masculinity stereotypes, and as surprise, which is a more neutral emotion.

Although the psychological and behavioral correlates of the emotion recognition biases observed in our study remain an open question for future investigations, they may reflect—beyond a general coping mechanism of avoiding negative emotion states associated with war trauma—gender stereotypes and social norms regarding emotional expression in men and women. It should be noted, however, that we could not investigate participant gender as a factor in our analysis, which may limit the generalizability of the results. In addition, in the absence of information about the target and the social context of the displayed emotions (Hess & Hareli, 2019), participants may have relied on subjective inferences about their meaning, which may have further influenced our results. Furthermore, stimuli were drawn from the Karolinska dataset, featuring White male and female faces, and may not fully match the facial phenotype of our West Asian White participants, potentially influencing their ability to identify emotional expressions. It should be noted that trauma exposure (i.e., HTQ‐I) was scored as either present or absent regardless of the frequency, intensity, or duration of exposure. Although this approach aligns with the original scoring procedures proposed by Mollica et al. (1992), the dichotomous scoring does not capture the severity or cumulative impact of repeated exposure to the same trauma type. This may result in an underestimation of individual differences in trauma burden, which should be addressed in future research using more nuanced measures of trauma exposure. Besides war‐related trauma, our participants may have also been exposed to other stressors related to migration and postmigration difficulties, which may compound their trauma severity compared to other civilian nondisplaced war survivors. Furthermore, research shows that a sense of new opportunities and life appreciation (i.e., posttraumatic growth) is not uncommon in trauma‐exposed individuals (Tedeschi & Calhoun, 2004). Future research should examine these factors with more gender‐balanced civilian populations exposed to war‐related trauma.

In conclusion, our study highlights the impact of war‐related trauma on emotional face processing, particularly in the recognition of negative emotions. By revealing emotion recognition deficits and biases that may reflect coping mechanisms and deeply ingrained social norms that shape how individuals perceive and respond to emotions in men and women in the context of adversity, our results suggest that emotion recognition may be an important skill on which to intervene to facilitate social adjustment in war trauma–exposed refugees.

## AUTHOR CONTRIBUTIONS

**Edita Fino**: Conceptualization; Methodology; Investigation; Writing – original draft; Writing – review and editing; Software; Formal analysis; Supervision; Validation. **Denis Mema**: Investigation; Writing – review and editing; Formal analysis; Software; Data curation. **Maria Ida Gobbini**: Writing – review and editing; Supervision; Validation.

## OPEN PRACTICES STATEMENT

The study reported in this article was not formally preregistered. Neither the data nor the materials have been made available on a permanent third‐party archive; requests for the data or materials can be sent via email to the lead author at edita.fino@unibo.it.

## Supporting information

Supporting Information
