# Supplementary material for: Impaired recognition of facial expressions of emotions in refugees: The role of war‐related trauma
Source: J Trauma Stress. 2025 Sep 14;39(1):35–43. doi: 10.1002/jts.70015 (PMC12890724; doi:10.1002/jts.70015)
Supplement: Supplementary file 1 — Supporting Information [file JTS-39-35-s001.docx]

Supplementary Material

**Figure S1.** Graphs show patterns of total errors in anger recognition (a), and per male and female faces (b).

**Figure S2.** Graphs show patterns of total errors in disgust recognition (a), and per male and female faces (b).

**Figure S3.** Graphs show patterns of total errors in fear recognition (a) and per male and female faces (b).

**Figure S4.** Graphs show patterns of total errors in sadness recognition (a), and per male and female faces (b).

**Figure S5.** Graphs show patterns of total errors in surprise recognition (a), and per male and female faces (b).

**Figure S6.** Graphs show patterns of total errors in joy recognition (a), and per male and female faces (b).
